# FAK inhibition radiosensitizes pancreatic ductal adenocarcinoma cells in vitro

**DOI:** 10.1007/s00066-020-01666-0

**Published:** 2020-07-23

**Authors:** A. Allam Mohamed, Andreas Thomsen, Marie Follo, Costantinos Zamboglou, Peter Bronsert, Hanan Mostafa, Aber Amen, Mohamed Mekawy, Anca L. Grosu, Thomas B. Brunner

**Affiliations:** 1 Department of Radiation Oncology, Medical Center, Faculty of Medicine, University of Freiburg, Freiburg, Germany; 2 Department of Medicine I, Medical Center, Faculty of Medicine, University of Freiburg, Freiburg, Germany; 3 Institute for Surgical Pathology, Medical Center, Faculty of Medicine, University of Freiburg, Freiburg, Germany; 4 Clinical Oncology and Nuclear Medicine Department, Assiut University Hospitals, Asyut, Egypt; 5 Partner Site Freiburg, German Cancer Consortium (DKTK), Freiburg, Germany; 6 Department of Radiation Oncology, RWTH Aachen University, Aachen, Germany; 7 Department of Radiation Oncology, University of Magdeburg, Magdeburg, Germany

**Keywords:** Pancreatic ductal adenocarcinoma, Focal adhesion kinase, Radiosensitization, Pancreatic stellate cell, Microenvironment, Stroma

## Abstract

**Introduction:**

Focal adhesion kinase (FAK) is a nonreceptor tyrosine kinase protein frequently overexpressed in cancer and has been linked to an increase in the stem cell population of tumors, resistance to therapy, and metastatic spread. Pharmacological FAK inhibition in pancreatic cancer has received increased attention over the last few years, either alone or in combination with other therapeutics including chemotherapy and immunotherapy. However, its prognostic value and its role in radioresistance of pancreatic ducal adenocarcinoma (PDAC) is unknown.

**Methods and materials:**

Using the TCGA and GTEx databases, we investigated the genetic alterations and mRNA expression levels of *PTK2* (the encoding-gene for FAK) in normal pancreatic tissue and pancreatic cancer and its impact on patient survival. Furthermore, we evaluated the expression of FAK and its tyrosine domain Ty-397 in three pancreatic cancer cell lines. We went further and evaluated the role of a commercial FAK tyrosine kinase inhibitor VS-4718 on the viability and radiosensitization of the pancreatic cell lines as well as its effect on the extracellular matrix (ECM) production from the pancreatic stellate cells. Furthermore, we tested the effect of combining radiation with VS-4718 in a three-dimensional (3D) multicellular pancreatic tumor spheroid model.

**Results:**

A database analysis revealed a relevant increase in genetic alterations and mRNA expression of the *PTK2* in PDAC, which were associated with lower progression-free survival*. *In vitro, there was only variation in the basal phosphorylation level of FAK in cell lines. VS-4718 radiosensitized pancreatic cell lines only in the presence of ECM-producing pancreatic stellate cells and markedly reduced the ECM production in the stromal cells. Finally, using a 3D multicellular tumor model, the combination of VS-4718 and radiotherapy significantly reduced the growth of tumor aggregates.

**Conclusion:**

Pharmacological inhibition of FAK in pancreatic cancer could be a novel therapeutic strategy as our results show a radiosensitization effect of VS-4718 in vitro in a multicellular 2D- and in a 3D-model of pancreatic cancer.

**Electronic supplementary material:**

The online version of this article (10.1007/s00066-020-01666-0) contains supplementary material, which is available to authorized users.

## Introduction

With a 5-year overall survival of less than 5%, pancreatic ductal adenocarcinoma is the 4th leading cause of cancer-related mortality in the western world and is expected to remain a leading cause of cancer-related mortality within the next decade due to the increasing incidence [1, 2]. Despite the grim prognosis, the relative pace of discovering novel treatments has been much slower than those for many other types of cancer.

Several reasons have been proposed to explain the poor prognosis. These include lack of specific symptoms for early disease diagnosis, the complexity of the molecular survival mechanisms, and the desmoplastic reaction (DR), which protects cancer cells against the therapy and facilitates their invasion into neighboring structures [3–5].

Clinically, pancreatic ducal adenocarcinoma (PDAC) is categorized into four stages: resectable, borderline-resectable, locally advanced and metastatic, based on the infiltration of the surrounding vascular structures and distant metastases [6]. While radiation therapy was shown to have little if any role in the first and last stages, the question of a potential benefit from radiotherapy in the borderline-resectable and locally advanced forms of the disease remains to be answered [7, 8]. Clinical studies have demonstrated a positive impact of fractionated chemoradiation [9] and stereotactic body radiotherapy (SBRT) [10] in advanced PDAC. However, it has been suggested that dose escalation is essential for this impact [11, 12]. Other studies did not conclude a benefit from adding chemoradiation in those stages [13]. Such uncertainty about the role of radiation therapy in PDAC could be attributed to the poor understanding of the molecular complexity of radioresistance mechanisms in pancreatic cancer. The interest in molecular targeted therapies in PDAC continues to increase. Here, we investigated the potential role of the focal adhesion kinase (FAK) in improving the radiosensitivity of PDAC.

FAK is a scaffolding nonreceptor tyrosine kinase, encoded by the *PTK2* gene (8q24.3). FAK is known to be involved in various functions within the cell, including cell–extracellular matrix (ECM) interactions [14], motility, anchorage-independent growth, migration [15, 16], proliferation, survival, and apoptosis [17], and expressed within the cancer stem cell (CSC) pool [18]. Structurally, it comprises three domains: an N‑terminal FERM (protein 4.1, ezrin, radixin, moesin) domain, a central kinase domain, and a c-terminal region containing proline-rich motifs and FAT (focal adhesion targeting). During activation, FAK undergoes conformational changes that result in the release of the bond between FERM and the kinase domains resulting in autophosphorylation of the Tyr397. Full activation of FAK occurs by the binding of Src to Tyr397 and the phosphorylation of other tyrosine domains within the activation loop (Tyr576 and 577) [19].

In the current study, we demonstrated alterations in the FAK encoding gene, *PTK2,* and mRNA expression using cBioPortal and the impact of those alterations on the prognosis. We then tested the effect of FAK inhibition in vitro on proliferation and the alteration of radiation sensitivity of cancer cell lines alone and in presence of stellate cells, using a *PTK2* siRNA and a commercially available potent FAK-tyrosine kinase inhibitor, VS-4718.

VS-4718 is an oral, selective FAK tyrosine kinase inhibitor (TKI) previously known as PND-1186. It has been tested in vitro and in vivo, and shows inhibition of tumor growth and spreading, selective depletion of the CSC pool and was found to potentiate the effect of other conventional chemotherapeutics such as paclitaxel and cisplatin [20–22]. To our knowledge, VS-4718 has not been tested until now for the radiosensitization of pancreatic cancer cells.

## Materials and methods

### *PTK2* gene alterations and expression analysis

*PTK2* gene alterations and mRNA in PDAC patients were queried online on cBioPortal for Cancer Genomics (http://www.cbioportal.org/) [23, 24] using the pancreatic adenocarcinoma (TCGA, PanCancer Atlas cohort) dataset. We queried mutations, putative copy-number alterations, and mRNA expressions (RNA Seq V2 RSEM with z‑scores = ±2) and survival analysis for the *PTK2* gene. We exported the data from the Oncoprint and survival screens. Differential expression of *PTK2 *between TCGA dataset and normal pancreatic tissue from Genotype-Tissue Expression (GTEx) [25] was queried using the GEPIA server [26]. *PTK2* expression in cancer cell lines was queried in the GEMicCCL portal [28].

### Cell lines and reagents

Human PDAC cell lines (PCC): Panc‑1, MIA PaCa 2 (American Type Culture Collection, Manassas, VA, USA) and PSN‑1 (Merck & Co., Inc., West Point, PA, USA). Human pancreatic stellate cell lines (h.PSCs) were kindly provided from Dr. J. Kleef’s Laboratory (Halle University, Germany), LTC-14 (immortalized rat pancreatic stellate cells) [29] were kindly provided by Dr. G. Sparmann (Rostock, Germany). Panc‑1, PSN‑1 and LTC-14 were grown in culture with DMEM and MIA PaCa 2 with RPMI 1640 + 10% fetal bovine serum (FBS) + 1% penicillin and streptomycin. Human PSCs were cultured in stellate cell medium: MDEM/F-12 and DMEM low glucose medium (1:1) + 16% FBS + 1% penicillin and streptomycin.

VS-4718 (Selleck Chemicals, Houston, TX, USA) was dissolved in dimethyl sulfoxide (DMSO); vehicle controls were equal volumes of the same concentration of DMSO.

### Western blotting

The antibodies used in the study: FAK antibody #3285, p‑FAK (Tyr397) antibody #3283 both at 1:1000 dilution (cell signaling, Cell Signaling Technology, Danvers, MA, USA), GAPDH and β‑actin at 1:10,000 dilution (Sigma-Aldrich, St. Louis, MI, USA). Cell lysate, protein separation, and immunoblotting were carried out as previously described [30].

### siRNA transfection

Cells were transfected according to the manufacturer’s instructions using the *PTK2*-siGENOME SMARTpool (Dharmacon, Lafayette, CO, USA) a mixture of four siRNA with the following target sequences:

- GCGAUUAUAUGUUAGAGAU
- GGGCAUCAUUCAGAAGAUA
- UAGUACAGCUCUUGCAUAU
- GGACAUUAUUGGCCACUGU

or ON-TARGET plus—nontargeting siRNA #2 (Dharmacon, Lafayette, CO, USA). Cells were incubated for 48 h with the transfection mixture and then used in experiments and to obtain cell lysate for Western blots.

### Cell viability assay

Cell viability and cytotoxicity were estimated using the MTT assay (3‑[4,5-dimethylthiazol-2-yl]-2,5-diphenyltetrazolium bromide) (Sigma-Aldrich, St. Louis, MI, USA). Cell lines (Panc‑1, PSN‑1, MIA PaCa 2 and LTC-14) were seeded in 96-well plates at a density between 1–5 × 10^4^ cells/well according to each cell line, and 24 h after incubation, the cells were treated with VS-4718 from 0–5 μM in the medium for 24 and 48 h. Cells were incubated with equal amounts of MTT (5 mg/ml) for 4 h in the dark at 37 °C. The supernatants were carefully discarded and replaced by 100 μl DMSO to dissolve the MTT in the cells. Finally, the optical density (OD) of each well was measured using an ELISA microplate reader at a wavelength of 570 nm. The results of each dose were normalized to the control group to calculate the viable fraction; each concentration was tested in triplicate and the mean was used and each experiment was repeated three times. The cytotoxicity of VS-4718 was expressed here as IC_50_ (which was estimated from a linear regression analysis of the experimental data).

### Clonogenic survival assay

Cancer cell lines were seeded directly into 6‑well plates as triplicates in monoculture settings. In the coculture assay, LTC-14 cells were seeded simultaneously with PCCs as they rapidly proliferate and adhere to the plates or hPSCs were seeded overnight before the PCCs to ensure they were adherent in the 6‑well plates with ratio 1:2–4 (PCCs: LTC14/hPSCs). For clonogenic survival assay on top of collagen I, 6‑well plates were coated with collagen G (Biochrom Inc., Germany).

Either VS-4718 (2.5 µM) or vehicle was added to the culture medium, then the 6 well-plates were incubated for 2 h and finally were irradiated using Cs-137, Gammacell 40 Exactor (Best Theratronics, Canada).

The culture medium was replaced 22 h after irradiation to wash out the treatment and plates were incubated at 37 °C and 5% CO_2_ for 10–14 days then fixed and stained using crystal violet.

Each experiment was repeated three times and results were plotted on a survival curve as mean ± standard deviation.

### Immunofluorescence labeling of γ-H2AX foci and cell cycle analysis

Three cell lines were co-cultured with PSCs as duplicates in two 96-well microplates, 190 µm Clear Bottom (Greiner Bio-one, Germany) and incubated together overnight at 37 °C to allow cells to attain growth until the mid-log phase before drug treatment. Cells were exposed to vehicle or VS-4718 (2.5 µM) for 1 h, later one of the microplates was irradiated with 6 Gy and then both microplates were incubated for 24 h at 37 °C and 5% CO_2._ Next, cells were fixed in cold methanol, membrane permeabilized using Triton X (Sigma, Germany) and incubated with Phospho-Histone H2AX (Ser139) Antibody #2577 at 1:100 dilution (Cell Signaling) and anti-Cytokeratin 8 antibodies #ab59400 at 1:100 dilution (Abcam) as primary antibodies. After which they were incubated with secondary antibodies, Alexa Fluor-488 goat anti-mouse and Alexa Fluor-546 goat anti-rabbit as 1:1000 dilution (Life Technologies, Thermo Fischer Inc., USA) and nuclear DNA was counterstained with DAPI. For cell cycle analysis, the microplates were coated with collagen type I (Collagen G, Biochrom Inc., Germany) before Panc‑1 cells were plated into wells as duplicates, to ensure activation of the β1-integrin-FAK axis [31], then treated with VS-4718 2.5 μM or vehicle for 24 h (exposure time for TKI in all radiation-sensitivity experiments). Later, cells were fixed with methanol at 20 °C and stained with DAPI to measure DNA content [32]. Both assays were assessed using high content microscopy (Olympus Scan*R, based on IX-81 inverted stage) and images were captured with a Hamamatsu Orca-ER and 20X UPLSAPO NA 0.75. Finally, the percentage of cancer cells with the number of foci above the basal average were represented in figures (Fig. 3 and suppl. Fig. 4a) as described before [33].

### Collagen content

Stellate cells were incubated in vitro for 5 days either with VS-4718 (2.5 µm) or with the control (DMSO) in 24-well plates in triplicate, then were fixed and stained with Picro Sirius red to detect the collagen content in the cell culture. Each experiment was repeated three times.

After repeated washing to remove the nonbound stain, we examined the stained tissue by phase-contrast and fluorescence microscopy. Later, we dissolved the Picro Sirius red using NaOH and measured OD at 540 nm wavelength of the dissolved solution to quantify the difference of collagen content.

### Three-dimensional multicellular tumor assay

We generated an in vitro multicellular three-dimensional (3D) tumor model using a standardized conical agarose microwell array (CAMA) platform as previously described [34]. CAMA allowed us to simultaneously generate hundreds of equally sized multicellular tumor aggregates for each matrix.

In short, agarose matrices were calibrated in cell culture medium 2 h before cell seeding. The cell suspension is used to seed an average of 50 cells per microwell; for coculture tumor spheroids the ratio of PSN‑1 and LTC-14 cells was 4:1, respectively. As a control, we also generated spheroids with only PSN‑1 and only LTC-14 (50 cells per microwell). The matrices containing tumor cells were incubated in 5% CO_2_ at 37 °C until they formed tumor aggregates on day 6, then they were treated with VS-4718 (2.5 μM) or vehicle. Then, 2 h later the matrices were irradiated with different doses (0–20 Gy, in 2 Gy gradient) in duplicate. The culture medium was replaced 24 h after the irradiation to wash out the treatment, and regularly every 3–4 days until day 28.

Volume-read out of tumor spheroids was done at day 6 and day 28 using a high-resolution scanner (CanoScan 9000F Mark II, Canon Inc.). Image processing and measuring the projected area of each cell aggregates was done using Image J software. We adopted spheroid control probability (SCP) and spheroid control dose 50 (SCD50) as analytical endpoints [35] on day 28. Cell aggregates were processed further for histology as previously described [34].

### Statistical analysis

Graphs were generated using Microsoft Excel 2016 and GraphPad Prism. Statistical analysis was done using an unpaired *t*-test. Data are presented as mean ± standard deviation (SD). Statistical significance was mentioned as follows: *:*P* < 0.05, **:*P* < 0.001 and ***:*P* < 0.0001.

## Results

### Overexpression and genetic alterations of FAK in PDAC negatively impact outcome

**Fig. 1:**
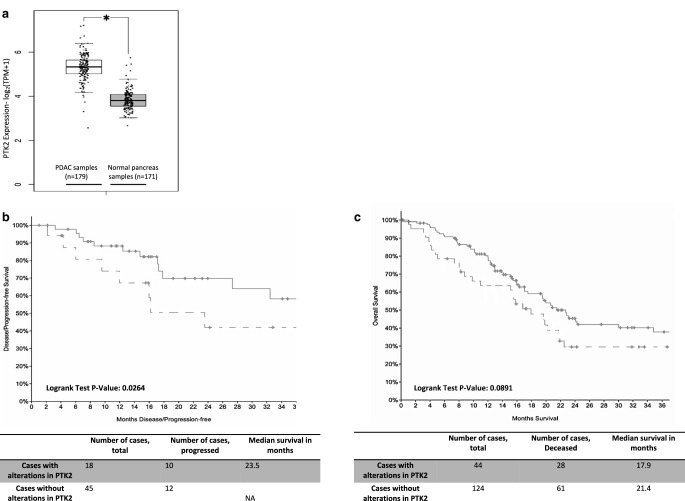
**a** Differential expression of *PTK2* between PDAC samples from TCGA (*white box*, *n* = 179) and normal pancreatic tissue (*grey box*, *n* = 171) from GTEx, data were mined from GEPIA and shown as a box plot (*t*-test; *p < 0.05). **b,c** Survival data from PanCancer Atlas cohort shown as Kaplan–Meier survival curves, 3‑year progression-free survival (PFS, **b**) and 3‑year overall survival (**c**), in patients with *PTK2* alterations; the *dotted lines* represent patients with any *PTK2 *alteration, and the *solid lines* represent patients without *PTK2* alterations. Log rank-test significant in 3‑year PFS (*p* = 0.0264) and nonsignificant in the 3‑year overall survival (*p* = 0.089)

First, we examined FAK differential expression between PDAC and normal tissue using GEPIA server. *PTK2 *expression was significantly higher in PDAC samples (*n* = 179) in comparison to the normal pancreatic tissue samples (*n* = 171) (Fig. 1a) and this overexpression was not significantly different across the four tumor stages (suppl. Fig. 1a).

Using cBioportal, we examined the TCGA, PanCancer atlas cohort, which included 168 pancreatic neoplasms samples with complete *PTK2* mutations and expression data from a total of 184 patients originally in the cohort. Alterations in the *PTK2* gene or its transcription were identified in 26% from PDAC samples (suppl. Fig. 1b).

The following alterations were identified: 15.48% high mRNA expression, 2.98% gene amplification, 0.6% mutation, deep deletion and low mRNA and 5.95% show multiple alterations. Amplification and gain were associated with the highest mRNA expression (suppl. Fig. 1c).

Using the survival data available from the cohort, we found that patients with any alteration in *PTK2 *gene had a significantly worse 3‑year progression-free survival and trend toward worse 3‑year survival in comparison to those without any *PTK2 *alteration (23.5 months vs not yet reached; *p*-value: 0.0264 and 17.9 vs 21.4 months; *p*-value: 0.0891, respectively; Fig. 1b,c).

### Effect of VS-4718 on the expression of FAK and pFAK in pancreatic cell lines

Using GEMiCCL portal, we queried *PTK2* in Panc‑1, PSN‑1 and MIA-PaCa 2. We did not find any *PTK2* mutations or a difference in the expression or copy-number variations between the cell lines (suppl. Table 1).

Using immunoblotting, the basal level of total FAK was similar in all three pancreatic cancer cell lines. However, the basal levels of p‑FAK (Y397) varied among them, with PSN‑1 expressing the highest basal level (suppl. Fig. 2a).

**Fig. 2:**
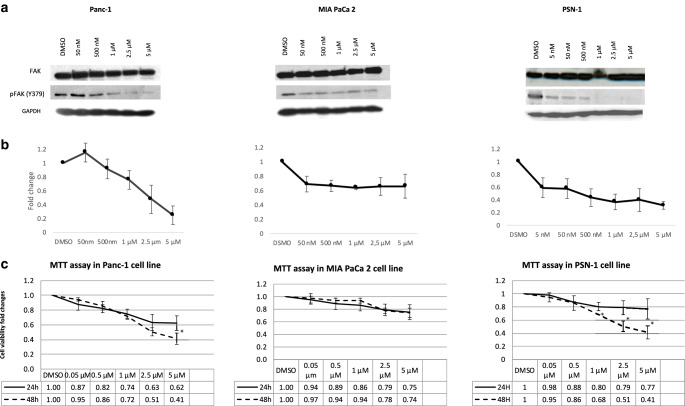
**a** Dose–gradient effect of VS-4718 on FAK Y397 dephosphorylation assessed 24 h in treated cells, GAPDH served as the loading control. **b** Densitometric analysis of p‑FAK (397) in three cell lines were semiautomatically calculated from VS-4718 treated cells normalized to DMSO using ImageJ; results represented as the mean (± standard deviation [SD]) of three experiments. **c** Cell viability was determined after 24 and 48 h of treatment with concentration-gradient of VS-4718 using MTT; data are shown as mean ± SD (*n* = 3) (*t*-test between 24 and 48 h values; *P < 0.05)

Next, we examined the effect of a VS-4718 dose-gradient on p‑FAK. There was a clear dose-dependent inhibition of p‑FAK in two cell lines (Panc‑1 and PSN-1), with a marked reduction in the p‑FAK at 2.5 µM in Panc‑1 and PSN‑1 starting from 0.5 µM. Meanwhile, after an initial reduction of p‑FAK in MIAPaca‑2, the effect was almost constant throughout the dosage range from 50 nM to 5 µM. (Fig. 2a,b).

### Effect of VS-4718 on cell growth of human pancreatic cancer cell lines

VS-4718 showed a dose-dependent reduction in all cell lines’ viability (Fig. 2c). The PSN‑1 cell line was the most sensitive cell line and the viability was significantly reduced with a longer exposure (24 h vs 48 h); the MIA PaCa 2 cell line again was the most resistant cell line to VS-4718. The IC_50_ doses for each cell line are shown in Table 1.

**Table 1:** VS-4718 IC_50_ in pancreatic cell lines

Cell line	IC_50_
Panc‑1	2.07 µM (95% CI 1.16–3.70 µM)
MIA PaCa 2	3.99 µM (95% CI 0.65–24.38 µM)
PSN‑1	1.23 µM (95% CI 0.54–2.81 µM)

Using single-cell suspensions from the Panc‑1 cell line with LTC-14 cultured for 5 days either with VS-4718 or vehicle in microwells, we noticed that Panc‑1 single cells were not able to form colonies in VS-4718 microwells. In control microwells they were able to form colonies surrounded by the stromal cells (LTC-14), which emphasizes continuous FAK inhibition with longer exposure of cells to VS-4718 has proliferation-inhibitory effects on cancer cells (suppl. Fig. 2b).

### VS-4718 in vitro sensitizes pancreatic cancer cells for radiation only as coculture with ECM-producing pancreatic stellate cells

**Fig. 3:**
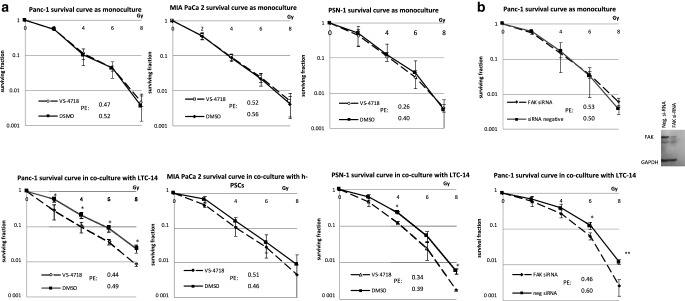
**a** VS-4718 increases PDAC radiation-sensitivity in a cell line dependent manner only as coculture with PSCs. Data shown as mean ± SD (*n* = 3; *t*-test; *P < 0.05, **P < 0.005). **b** FAK knockdown reproduced the same pattern of radiosensitization of PDAC cells only as coculture. Data shown as mean ± SD (*n* = 3; *t*-test; *P < 0.05). *PE* plating efficiency

While VS-4718 did not sensitize any of the three cell lines for radiation as a monoculture, using in vitro coculture set up for the assay by seeding single-cell suspensions from cancer cells on top of cultured human stellate cells or simultaneously with the LTC-14 immortalized stellate cells, VS-4718 had a significant effect on radiosensitization in at least two cell lines (Panc‑1 and PSN-1), and a statistically insignificant effect in the MIA PaCa 2 cell line (Fig. 3a).

To confirm that the radio-sensitizing mechanism of VS-4718 was attributed to FAK, we repeated the clonogenic assay using siRNA to knockdown *PTK2*. The results confirmed the same pattern of radio-sensitization only in the presence of stellate cells as an in vitro coculture (Fig. 3b). The radiosensitizing effect was similar using the h.PSCs or LTC-14 as stellate cells in the coculture (suppl. Fig. 3b,c) Also, the pharmacological FAK inhibition radiosensitized Panc‑1 on top of collagen I layer (suppl. Fig. 3d). The previous results emphasize the role of the ECM-producing stellate cells to stimulate FAK-derived survival in cancer cells and the interception of this axis would increase their radiosensitivity.

### VS-4718 impaired DNA-repair in pancreatic cancer cells and arrested cell cycle

To examine how FAK inhibition could alter the radiosensitivity, we measured the level of residual γ‑H2AX foci in cancer cells 24 h after radiation which correlates with the impairment of DNA repair and radiosensitivity of the irradiated PCCs in coculture with PSCs. A level of 5–7 foci per nucleus was considered as the lower cut off, depending on the basal level of γ‑H2AX foci for cell lines as mentioned before [33].

**Fig. 4:**
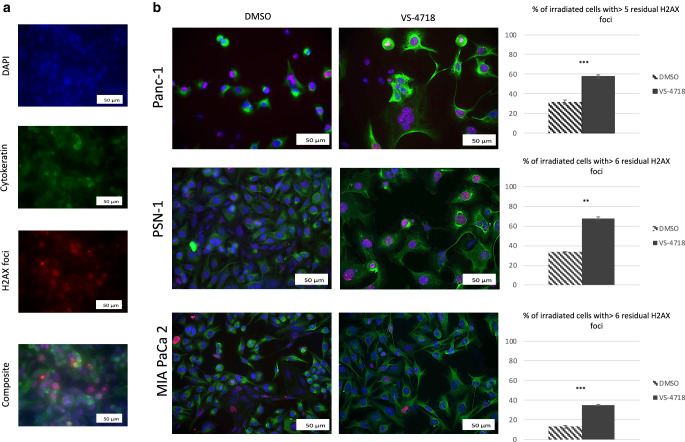
**a** Effect of VS-4718 on γ‑H2AX foci in Panc‑1 cell line in coculture with PSCs 24 h after radiation (VS-4718 2.5 µM was added 1 h before 6 Gy radiotherapy), cells were fixed labeled against γ‑H2AX, nuclei were stained with DAPI and PCCs were labeled against cytokeratin 8, then images were taken at 20 × objective, scale bar = 50 µm, finally a composite from three images (DAPI, cytokeratin‑8, and γ‑H2AX) was generated using ImageJ. **b** Composite images from three cell lines with bars represent the percentage of cancer cells with more than 5 or 6 foci per nucleus, scale bar = 50 µm. Data shown as mean ± standard deviation, *n* = 3; *t*-test; **P < 0.01, ***P < 0.001

We found a significant increase in the percentage of cancer cells having a significant number of foci using VS-4718 and radiotherapy in comparison to radiotherapy alone (Fig. 4). VS-4718 alone did not increase the number of γ‑H2AX foci, indicating that inhibiting FAK impaired DNA repair only (suppl. Fig. 4a).

Further, Panc‑1 exposure to 2.5 µM of VS-4718 for 24 h (inhibitor-exposure time of cell lines in radiation-sensitivity assays) resulted in cells arrest in the radiation-sensitive G2/M phase of the cell cycle 38.76 ± 7.53% vs. 10.16 ± 4.02% in control (DMSO) (suppl. Fig. 4b).

### VS-4718 reduced the proliferation of tumor stromal cells and ECM production in vitro

**Fig. 5:**
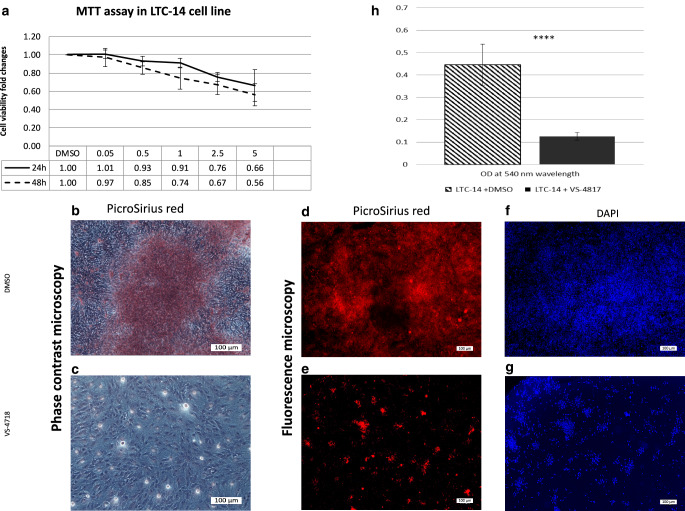
**a** Inhibitory effect of VS-4718 on LTC-14 cell line determined by MTT assay, data are shown as mean ± SD (*n* = 3). **b** PicroSirius red staining for collagen content produced by stellate cells (LTC-14 cell line) under Phase contrast microscopy of LTC-14 cells; for control (DMSO) strongly stained and **c** VS-4718 (2.5 µM) faintly stained, were taken at 10 X objective, scale bar 100 µm. The same experiment was repeated under fluorescence microscopy, PicroSirius red staining for collagen content was strongly reduced under the inhibitory effect of VS-4718 2.5 (2.5 µM) (**e**) in comparison to the DMSO (**d**). Furthermore, the proliferation of LTC-14 was strongly inhibited under VS-4718 as seen in DAPI staining of the nuclei (**g**) in comparison to DMSO (**f**), images were taken at 4X objective, scale bar 100 µm

We examined the impact of FAK inhibition using VS-4718 on stellate cells as the main component of the tumor microenvironment. VS-4718 reduced the viability of stellate cells (LTC-14) in a dose-dependent manner (Fig. 5a).

In addition, VS-4718 significantly inhibited the collagen production of stellate cells in vitro, as indicated by Picro Sirius red staining, under both phase contrast and fluorescence microscopy (Fig. 5b–g). We dissolved the attached staining and measured its OD to quantify this effect, which revealed a marked reduction of collagen content (Fig. 5h).

### Combination of radiotherapy and VS-4718 effectively inhibited multicellular tumor spheroid growth

**Fig. 6:**
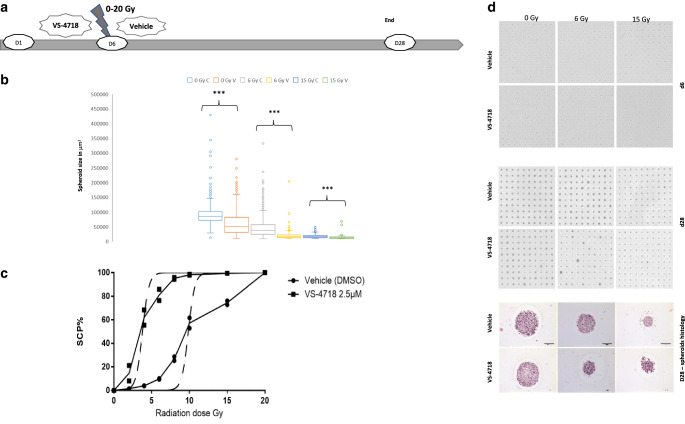
**a** Timeline of the multicellular experiment; *d0*: cell mixture (PSN-1 + LTC + 14) seeding in the matrices, *d6*: treatment with VS-4718 or control then irradiation, finally *d28*: the final readout of the experiment and harvesting cellular aggregates for histology. **b** Box plot represents the means ± standard deviation (SD) of spheroids size-read out d28 at three radiation dose points: 0, 6, 15 Gy with VS-4718 (V) or control (C) respectively(*light blue*: 0 Gy + control, *orange*: 0 Gy + VS-4718, *grey*: 6 Gy + control, *yellow*: 6 Gy + VS-4718, *dark blue*: 15 Gy + control, *green*: 15 Gy + VS-4718). Each bar represents an average of 400 spheroids (*n* = 2; *t*-test; ***P < 0.001). VS-4718 alone significantly reduced spheroid size, and the inhibition was stronger in association with radiation. **c** The dose–response curve of PSN-1 + LTC-14 spheroids: the two *solid curves* represent the probability of inhibition of spheroid’s growth spheroids control probability (SCP%), while the two *dashed vertical lines* represent the 95% confidence intervals of the SCD_50_. **d** Scans of multicellular spheroids (PSN-1 + LTC-14) in the conical agarose microwell array (CAMA) on d6 and d28 at three dose-points 0, 6, and 15 Gy, with H&E histology of the multicellular aggregates at d 28, The synergistic difference of the therapy in spheroids’ size appears clearly at 6 Gy

We examined the hypothesis of FAK modulation of radiation sensitivity in a 3D multicellular model (PSN-1 + LTC-14) that mimics to a greater extent a solid tumor compared to the 2‑dimensional monolayer coculture. VS-4718 alone reduced the size of spheroids but could not produce spheroid control (complete inhibition of spheroid growth; Fig. 6b). Also, irradiation alone resulted in the size reduction of the multicellular aggregates (Fig. 6b) but the spheroid control could not be achieved unless high doses were used. However, the additive effect of combined therapy effectively inhibited spheroid growth at lower radiation doses (Fig. 6d). We generated a spheroid control probability (SCP) curve vs. radiation dose, using a radiation dose range from 0 to 20 Gy (Fig. 6c). The tumor-stromal aggregates were completely controlled (100% nongrowing) after 20 Gy, this effect was achievable at half of the radiation dose (10 Gy) with the combined therapy. The SCD_50_ was 9.8 and 3.8 Gy for radiation alone and radiation + VS-4718, respectively, indicating the strong radiosensitizing effect of FAK inhibition in vitro in the 3D multicellular tumor model.

## Discussion

PDAC represents a major challenge based on the scarcity of effective therapy. Local progression alone can be the leading cause of death in 30% of the patients [36]. The desmoplastic reaction and the interactions between tumor cells and ECM have been identified as being central to the process of PDAC progression and resistance to standard therapies [37]. Stellate cells are the primary source of ECM production in PDAC, being activated in carcinogenesis and showing increased alpha-SMA expression after chemoradiation [38], which may indicate a role in the therapy resistance of PDAC.

ECM induces integrin activation and assembly of focal adhesions with phosphorylation of FAK in cancer cells, which in turn potentiates the proliferation, stress survival, cancer stemness and migration [31]. While FAK has been considered lately valuable druggable target in cancer therapy, results from phase I studies mostly indicating stationary course as the best clinical outcome under FAK inhibitors [39]. This is important because it indicates that FAK inhibition would effectively work as a combination. Indeed, FAK inhibition has been found to improve the radiation sensitivity in HNSCC [40], KRAS mutant NSCLC [41], and glioblastoma multiforme [42].

In the current study, we found that FAK was significantly overexpressed in PDAC. Although its mutational incidence was very low, other genetic alterations, including mRNA expression, were high (26%), and this significantly reduced the progression free survival and may also reduce the overall survival in those patients. To our knowledge, we are the first to report this prognostic fact in PDAC.

For pharmacological FAK inhibition in this study, we used the commercially available tyrosine kinase inhibitor VS-4718, which is a highly selective reversible small-molecule FAK inhibitor. When using VS-4718 in the µM range, only FAK and FLT3 have negligible activity with the latter expressed only in cells of hematopoietic origin [43]. The IC_50_ of VS-4718 in pancreatic cancer cell lines ranged from 1.23 to 3.99 µM, whereby the response between the three cell lines to VS-4718 varied, which is in agreement with the findings of Shapiro et al., who reported fluctuations in IC_50_ of VS-4718 in different cell lines, having attributed it to the difference in Merlin’s expression which regulates FAK function [44], and also with findings in pediatric tumor cell lines [45]. The pharmacological inhibition of FAK could be improved further, especially in the MIA PaCa 2 cell line if higher doses were used. Furthermore, we used siRNA to knockdown *PTK2* as a complementary method to alleviate the concerns that current results of VS-4718 could be due to off-target effects.

The presence of ECM-producing stellate cells in the milieu or ECM (collagen I) were an essential component that allowed the radiation-sensitizing effect of FAK inhibition to take place in a cell-line-dependent manner. These findings align with the findings from Mantoni et al. [46], who reported for the first time the potential radioprotective role of the stellate cells. Mantoni et al. attributed the effect to heterotypic signaling, which was found to be due to β1-integrin activation through ECM production. Also, our findings confirm data previously reported by Begum et al. about the role of a collagen I effect in the activation of FAK and intercepting this axis through pharmacological FAK inhibition resulted in a reduction of clonogenic PDAC growth and migration, and extended antitumor response to other therapeutics as gemcitabine and nab-paclitaxel [31]. Our study also indicates that FAK may be essential in DNA damage response and its pharmacological inhibition resulted in the maintenance of DNA damage response after radiation. Indeed, it has been reported that FAK-deficient breast cancer cells (PyMT) experienced arrest in cell growth and underwent apoptosis [47]. Further analysis revealed that the pharmacological FAK inhibition blocks the cell cycle in the G2/M phase, resulting in improved radiation sensitivity.

VS-4718 also effectively targeted the stellate cells, reduced their viability, and markedly reduced the ECM content in the culture, which would generally contribute to a better response of cancer cells to therapy. This has been previously reported using VS-4718 in PDAC in vivo resulting in the improvement of tumor response to checkpoint immunotherapy [20].

To address the shortcoming of not using an in vivo model, we subsequently adopted the CAMA platform to generate a multicellular (cancer and stellate cells) 3D model of PDAC. The pros of this platform include the following: the reproducible production of hundreds of tumor aggregates with a similar average number of cells and size; an in vitro 3D multicellular model that allows the examination of the relationship between specific components of the tumor microenvironment; and it acts as a standardized platform to test new therapies. Lastly, the conical design of the CAMA allows the multicellular tumor aggregates to grow as a single aggregate per microwell, independent from the spheroid-forming ability of the cancer cells [34]. The cons are mostly limited to the large design of the experiment using this platform to reach accurate readout, which would require a large number of matrices, cells and 6-wells plates, which limited our 3D-model experiment to only one cell line (PSN-1 with LTC-14).

We found the endpoints SCP and SCD_50_, mentioned by Ingargiola et al. [35], useful to evaluate the effect of therapy in our 3D spheroid model. We were able to scan the size of an average of 400 tumor aggregates per matrix along the radiation-dose gradient from 0–20 Gy in each arm of the experiment. That gave us more confidence about the interpretation of the entire experiment in comparison to other methods that allow the generation of only a few spheroids per experiment and where the spheroids are manually evaluated. In our hands, the inhibitory effect on the growth of the multicellular tumor aggregates between VS-4718 and radiation vs. radiation alone was undoubtedly strong, and SCD_50_ was 3.8 vs 9.8 Gy, respectively.

Taken together, pharmacological FAK-targeting strategies in PDAC, here VS-4718, may provide a therapeutic option for a certain subset of patients in the era of personalized therapy. Our experiments provide evidence for the in vitro radiosensitizing effect of VS-4718 in multicellular 2D and 3D models of PDAC. With the advancement of irradiation techniques in preclinical models, our data will open the door in the future to allow the evaluation of the radiosensitizing effect of FAK inhibition in vivo [48].

## Caption Electronic Supplementary Material

Suppl. Tab. 1: Level of PTK in the cell lines

Suppl. Fig. 1: **a** PTK2 mRNA expression in PDAC among different tumor stages, (ANOVA test). **b–c** OncoPrint screens from CbioPortal illustrating the genetic alterations and expression heatmap in TCGA, PanCancer dataset. **d** represents PTK2 gene alterations versus mRNA expression from the dataset Pancreatic Adenocarcinoma (TCGA, PanCancer). PTK2 amplification and gain associated mostly with increase in mRNA expression. Suppl. Fig. 2: **a** Western blot of the basal expression of FAK and pFAK in 3 pancreatic cancer cell lines (*n* = 3). **b** Using single cell suspensions from the Panc‑1 cell line with LTC-14 cultured for 5 days either with VS-4718 or vehicle in microwells, cells were fixed, cancer cells labeled against cytokeratin 8. VS-4718 inhibited cell proliferation and Panc 1 cells couldn’t form colonies after 5 days of incubation (1), while they were able to proliferate and multiply in control microwells (2). PCCs were labeled against cytokeratin 8, then images were taken at 4x objective, scale bar  = 500 μm. (not quantitively analyzed). Suppl. Fig. 3: **a–d** VS-4718 without stellate cells has no radio-sensitization effect at 6 Gy on Panc‑1 cell alone (**a**) the radio-sensitization effect appears only in co-culture with PSCs (no difference between h‑PSCs (**b**) or LTC-14 (C) ) also the effect appears by culturing Panc‑1 on top of collagen‑I layer (**d**). Altogether, FAK-inhibition block the survival stimulation of ECM to tumor cells. Data show as mean ± SD (*n* = 3; *t*-test; *P < 0.05) **e** Tumor cell colonies from a clonogenic survival assay with PCCs (Panc-1) in coculture with PSCs. Suppl. Fig. 4: **a** Effect VS-4718 on γ‑H2AX foci in 3 cancer lines in coculture with PSCs after 24h exposure (without irradiation), cells were fixed and stained for γ‑H2AX, cancer cells were labeled against cytokeratin 8, bars represent the percentage of cancer cells with more than 5 or 6 foci per nucleus. VS-4718 alone doesn’t induce increases in γ‑H2AX foci compared to control. **b** Treatment of Panc‑1 cells on collagen I layer with VS-4718 resulted in G2/M arrest. Summary of percentage of cells in each cell cycle phase after the treatment of Panc‑1 cells with VS-4718 for 24 h. Data is shown as the % of cells in G1, S, and G2‑M phases ± SD. (*n* = 3; *p <0.05)
