# Supplementary material for: FAK inhibition radiosensitizes pancreatic ductal adenocarcinoma cells in vitro
Source: Strahlenther Onkol. 2020 Jul 23;197(1):27–38. doi: 10.1007/s00066-020-01666-0 (PMC7801360; doi:10.1007/s00066-020-01666-0)
Supplement: Supplementary file 1 — Suppl. Tab. 1: Level of PTK in the cell lines [file 66_2020_1666_MOESM1_ESM.docx]

**Suppl. Table-1**

|  | **Panc-1** | **PSN-1** | **MIA Paca2** |
| --- | --- | --- | --- |
| Level of *PTK2* expression in CCLE | 53.71% | 52.48% | 53.55% |
| PTK2 copy-number  In COSMIC | Not available | 3 | 3 |

**Supplementary figures**

Figure I

I-a: *PTK2* mRNA expression in PDAC among different tumor stages, (ANOVA test).

1-b-c: OncoPrint screens from CbioPortal illustrating the genetic alterations and expression heatmap in TCGA, PanCancer dataset.

1-d represents PTK2 gene alterations versus mRNA expression from the dataset Pancreatic Adenocarcinoma (TCGA, PanCancer). *PTK2* amplification and gain associated mostly with increase in mRNA expression.

Figure II

2-a: Western blot of the basal expression of FAK and pFAK in 3 pancreatic cancer cell lines (n=3).

2-b Using single cell suspensions from the Panc-1 cell line with LTC-14 cultured for 5 days either with VS-4718 or vehicle in microwells, cells were fixed, cancer cells labeled against cytokeratin 8. VS-4718 inhibited cell proliferation and Panc-1 cells couldn’t form colonies after 5 days of incubation (1), while they were able to proliferate and multiply in control microwells (2). PCCs were labeled against cytokeratin 8, then images were taken at 4x objective, scale bar =500 µm. (not quantitively analyzed)

Figure III:

3-a-d: VS-4718 without stellate cells has no radio-sensitization effect at 6 Gy on Panc-1 cell alone (a) the radio-sensitization effect appears only in co-culture with PSCs (no difference between h-PSCs (b) or LTC-14 (C) ) also the effect appears by culturing Panc-1 on top of collagen-I layer (d). Altogether, FAK-inhibition block the survival stimulation of ECM to tumor cells. Data show as mean ± SD (n = 3; t test; * P < 0.05)

3-e: Tumor cell colonies from a clonogenic survival assay with PCCs (Panc-1) in coculture with PSCs.

Figure IV:

4-a: Effect VS-4718 on γ-H2AX foci in 3 cancer lines in coculture with PSCs after 24 hours exposure (without irradiation), cells were fixed and stained for γ-H2AX, cancer cells were labeled against cytokeratin 8, bars represent the percentage of cancer cells with more than 5 or 6 foci per nucleus. VS-4718 alone doesn’t induce increases in γ-H2AX foci compared to control.

4-b: Treatment of Panc-1 cells on collagen I layer with VS-4718 resulted in G2/M arrest. Summary of percentage of cells in each cell cycle phase after the treatment of Panc-1 cells with VS-4718 for 24 h. Data is shown as the % of cells in G1, S, and G2-M phases ± SD. (n=3, * p <0.05)
