# Supplementary material for: FAK inhibition radiosensitizes pancreatic ductal adenocarcinoma cells in vitro
Source: Strahlenther Onkol. 2020 Jul 23;197(1):27–38. doi: 10.1007/s00066-020-01666-0 (PMC7801360; doi:10.1007/s00066-020-01666-0)
Supplement: Supplementary file 2 — Suppl. Fig. 1: a PTK2 mRNA expression in PDAC among different tumor stages, (ANOVA test). b–c OncoPrint screens from CbioPortal illustrating the genetic alterations and expression heatmap in TCGA, PanCancer dataset. d represents PTK2 gene alterations versus mRNA expression from the dataset Pancreatic Adenocarcinoma (TCGA, PanCancer). PTK2 amplification and gain associated mostly with increase in mRNA expression. Suppl. Fig. 2: a Western blot of the basal expression of FAK and pFAK in 3 pancreatic cancer cell lines (n = 3). b Using single cell suspensions from the Panc‑1 cell line with LTC-14 cultured for 5 days either with VS-4718 or vehicle in microwells, cells were fixed, cancer cells labeled against cytokeratin 8. VS-4718 inhibited cell proliferation and Panc 1 cells couldn’t form colonies after 5 days of incubation (1), while they were able to proliferate and multiply in control microwells (2). PCCs were labeled against cytokeratin 8, then images were taken at 4x objective, scale bar = 500 μm. (not quantitively analyzed). Suppl. Fig. 3: a–d VS-4718 without stellate cells has no radio-sensitization effect at 6 Gy on Panc‑1 cell alone (a) the radio-sensitization effect appears only in co-culture with PSCs (no difference between h‑PSCs (b) or LTC-14 (C) ) also the effect appears by culturing Panc‑1 on top of collagen‑I layer (d). Altogether, FAK-inhibition block the survival stimulation of ECM to tumor cells. Data show as mean ± SD (n = 3; t-test; *P < 0.05) e Tumor cell colonies from a clonogenic survival assay with PCCs (Panc-1) in coculture with PSCs. Suppl. Fig. 4: a Effect VS-4718 on γ‑H2AX foci in 3 cancer lines in coculture with PSCs after 24h exposure (without irradiation), cells were fixed and stained for γ‑H2AX, cancer cells were labeled against cytokeratin 8, bars represent the percentage of cancer cells with more than 5 or 6 foci per nucleus. VS-4718 alone doesn’t induce increases in γ‑H2AX foci compared to control. b Treatment of Panc‑1 cells on collag [file 66_2020_1666_MOESM2_ESM.pdf]

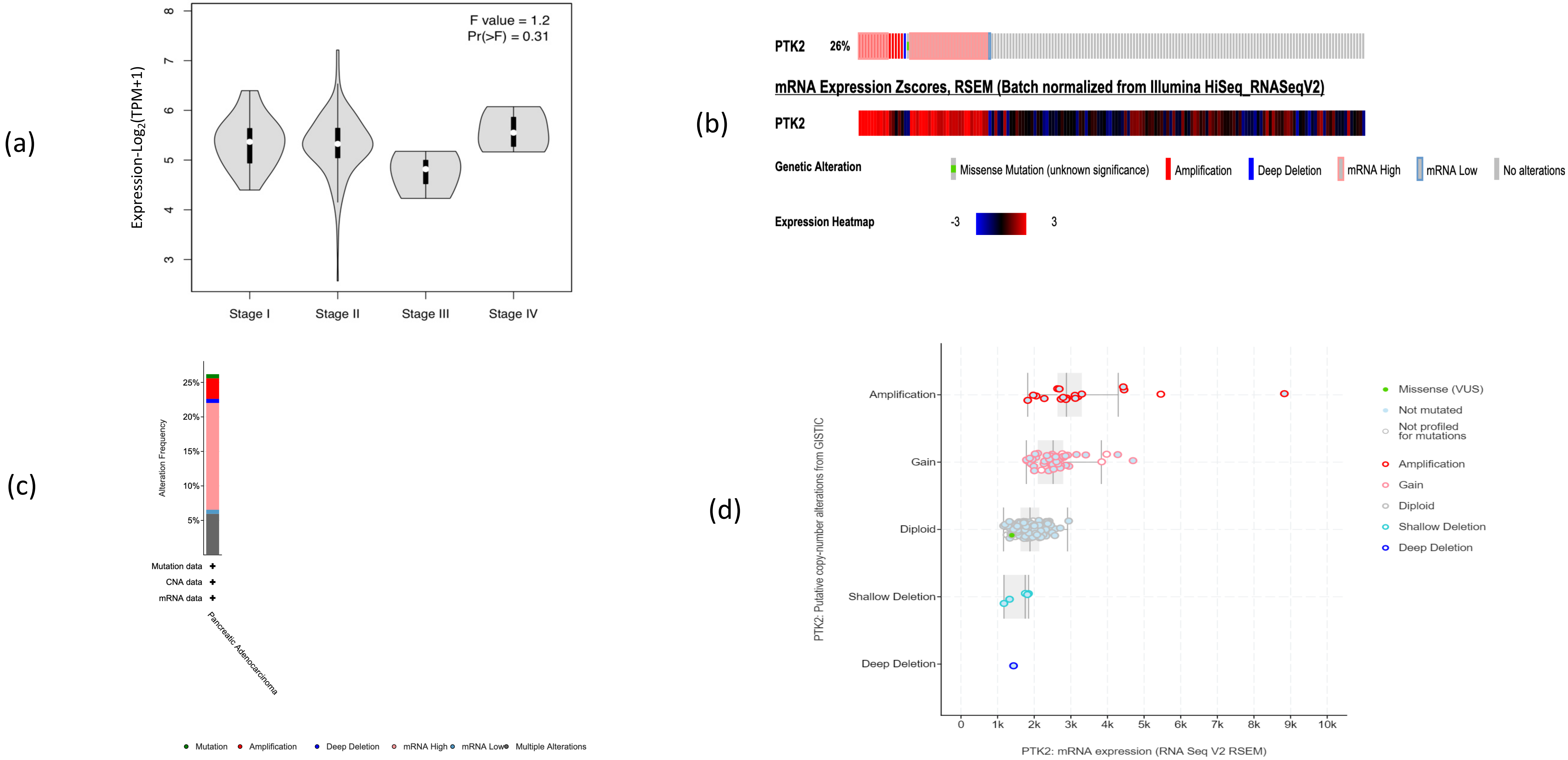
(a)
F value = 1.2
Pr(>F) = 0.31
Expression-Log2(TPM+1)
Stage I
Stage II
Stage III
Stage IV
(b)
PTK2
26%
mRNA Expression Zscores, RSEM (Batch normalized from Illumina HiSeq_RNASeqV2)
PTK2
Genetic Alteration
Missense Mutation (unknown significance)
Amplification
Deep Deletion
mRNA High
mRNA Low
No alterations
Expression Heatmap
-3
3
(c)
Alteration Frequency
25%
20%
15%
10%
5%
Mutation data
CNA data
mRNA data
Pancreatic Adenocarcinoma
Mutation
Amplification
Deep Deletion
mRNA High
mRNA Low
Multiple Alterations
(d)
PTK2: Putative copy-number alterations from GISTIC
Amplification
Gain
Diploid
Shallow Deletion
Deep Deletion
0
1k
2k
3k
4k
5k
6k
7k
8k
9k
10k
PTK2: mRNA expression (RNA Seq V2 RSEM)
Missense (VUS)
Not mutated
Not profiled for mutations
Amplification
Gain
Diploid
Shallow Deletion
Deep Deletion

(a)

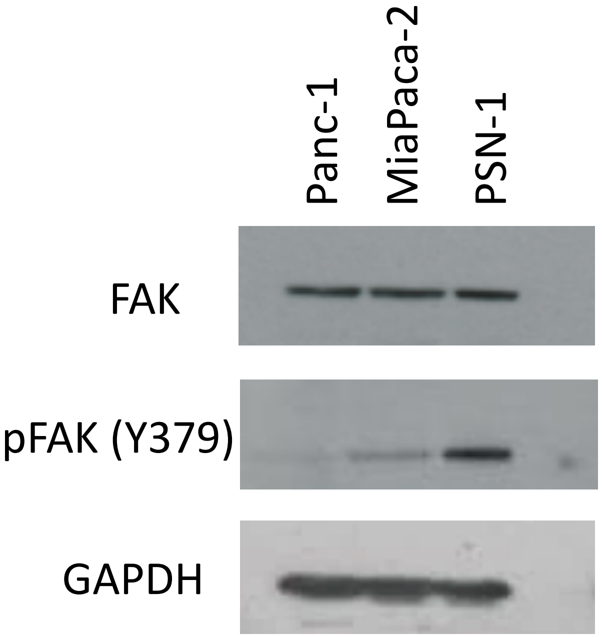

(b)

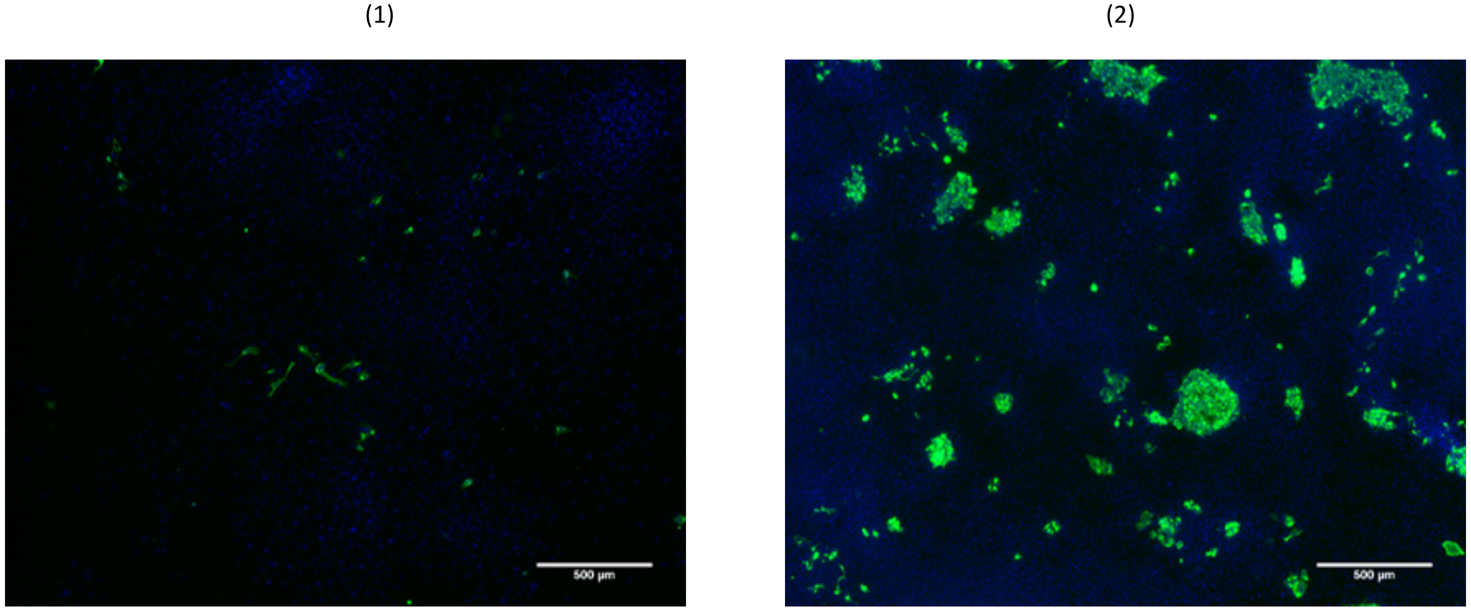

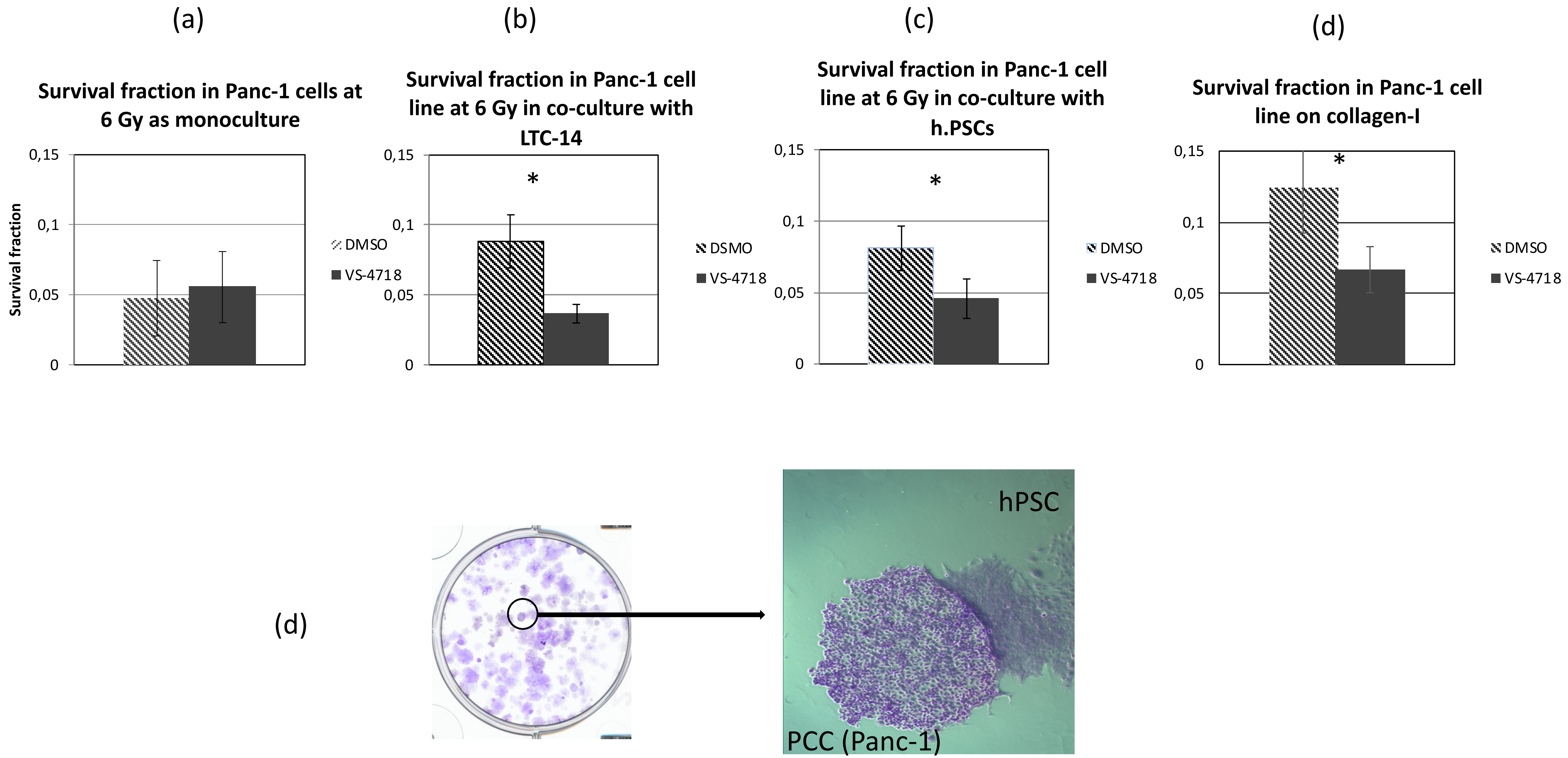
(a)
Survival fraction in Panc-1 cells at 6 Gy as monoculture
Survival fraction
0,15
0,1
0,05
0
DMSO
VS-4718
(b)
Survival fraction in Panc-1 cell line at 6 Gy in co-culture with LTC-14
*
DSMO
VS-4718
(c)
Survival fraction in Panc-1 cell line at 6 Gy in co-culture with h.PSCs
*
DMSO
VS-4718
(d)
Survival fraction in Panc-1 cell line on collagen-I
*
DMSO
VS-4718
(d)
hPSC
PCC (Panc-1)

(a)

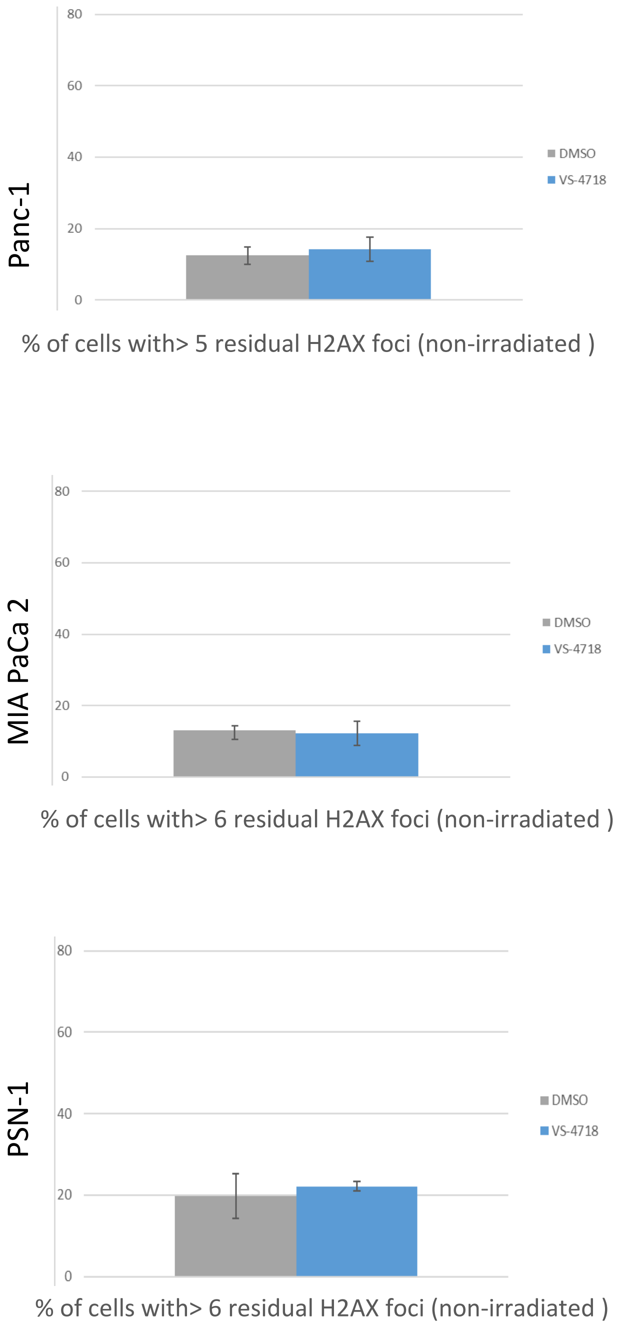
Panc-1
80
60
40
20
0
DMSO
VS-4718
% of cells with> 5 residual H2AX foci (non-irradiated)
MIA PaCa 2
80
60
40
20
0
DMSO
VS-4718
% of cells with> 6 residual H2AX foci (non-irradiated)
PSN-1
80
60
40
20
0
DMSO
VS-4718
% of cells with> 6 residual H2AX foci (non-irradiated)

(b)

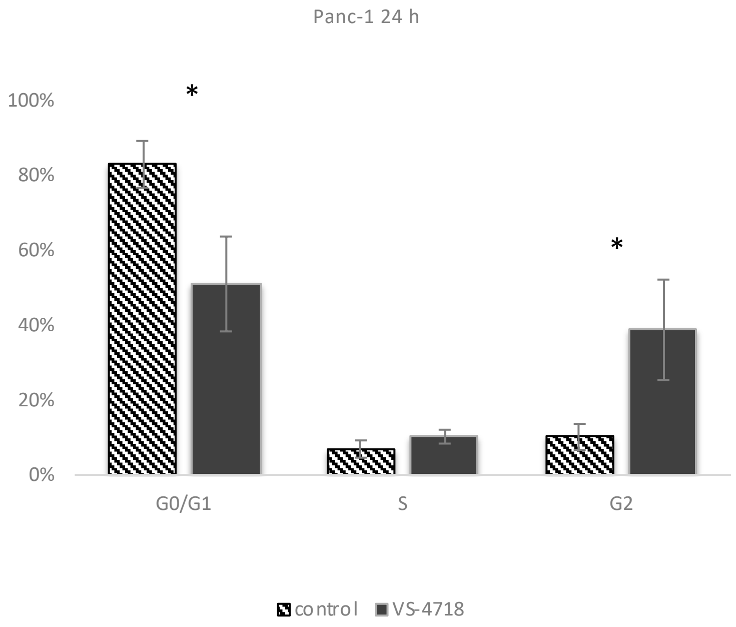
Panc-1 24 h
100%
80%
60%
40%
20%
0%
*
*
G0/G1
S
G2
control
VS-4718
